# Patterns and Determinants of Antibiotic Use Behaviors among Rural Community Residents in Eastern China

**DOI:** 10.3390/antibiotics11060823

**Published:** 2022-06-18

**Authors:** Yanhuan Wang, Xinping Zhao, Yurong Li, Na Wang, Feng Jiang, Helen Lambert, Fei Yan, Chaowei Fu, Qingwu Jiang

**Affiliations:** 1 Key Laboratory of Public Health Safety, NHC Key Laboratory of Health Technology Assessment, School of Public Health, Fudan University, Shanghai 200032, China; 20211020022@fudan.edu.cn (Y.W.); xpzhao@shmu.edu.cn (X.Z.); 19211020059@fudan.edu.cn (Y.L.); na.wang@fudan.edu.cn (N.W.); jf98@fudan.edu.cn (F.J.); fyan@shmu.edu.cn (F.Y.); jiangqw@fudan.edu.cn (Q.J.); 2 Population Health Sciences, Bristol Medical School, University of Bristol, Bristol BS8 2PS, UK

**Keywords:** antibiotic use, antibiotic access, determinants, community population, rural China

## Abstract

Inappropriate antibiotic use may lead to antibiotic resistance, which has become a serious global crisis. Addressing suboptimal antibiotic use in the general population can play a significant role in the fight against antimicrobial resistance. This study aims to describe antibiotic use and sources of acquisition, and to identify factors influencing antibiotic access among rural community residents in Eastern China. A cross-sectional survey was conducted from July to August 2020, and 1494 participants from two villages in Eastern China were enrolled. Information was obtained using face-to-face interviews with a structured electronic questionnaire. Chi-squared and multinominal logistic regression analysis were used to explore possible determinants. In total, 1379 participants were eligible for the analysis. In the past 12 months, nearly half the respondents had taken any antibiotic (48.4%), and this proportion varied across marital status and age group. Two thirds of them (59.9%) obtained antibiotics from medical facilities with a prescription when they last took antibiotics, while 17.7% and 22.4% chose retail pharmacies and other sources, respectively. Multinominal analysis found that a higher proportion obtained antibiotics outside medical facilities among those aged 15 to 44 years, unmarried, non-white collar workers, with more years of education, lower annual household income per capita and lower levels of antibiotic knowledge. The antibiotic use behavior of rural community residents in Eastern China remains suboptimal. Antibiotic use and access behaviors need to be further addressed. Effective antibiotic stewardship in non-medical facility sources and training programs targeted for rural Chinese is warranted in future.

## 1. Introduction

Antimicrobial resistance (AMR) poses a major threat to human health around the world, which is a serious problem, especially in middle- and low-income countries including China [1,2], and has been paid increasing attention in recent years [3]. The cost of AMR is immense, both economically as well as for human lives [4]. It is estimated that deaths attributable to AMR will reach 4.73 million in Asia and 10 million worldwide by 2050, and that global GDP will shrink by 2.5–3% if the current problem of antibiotic resistance is not alleviated [5]. One of the most important contributors to this global crisis is the inappropriate use of antibiotics in human health [6]. In China, a systematic review [7] showed that over 80% of outpatients with upper respiratory infections were being prescribed antibiotics, and high rates of unnecessary antibiotic use are associated with increasing rates of AMR in hospitals and the environment [8].

The spread of AMR is an urgent issue, requiring global and coordinated efforts to address [9,10,11]. In 2011 the World Health Organization (WHO) proposed a global action plan on AMR to limit resistance and called for judicious use of antimicrobial drugs [12]. Another global action was endorsed at the World Health Assembly in 2015 to tackle the growing problem of AMR [11]. Additionally, world leaders at the G20 Summit agreed to promote prudent use of antibiotics in 2016 [13]. However, the capacity for AMR control varies over countries and regions worldwide. Some WHO member states, including European countries and the United States, have released their own strategies and action plans on AMR in recent years [3]. In the past two decades, the Chinese government has made appreciable efforts against AMR, such as the establishment of the Center for Antibacterial Surveillance in 2005 to monitor clinical antibiotic use [14], and the implementation of the Administrative Measures for Clinical Use of Antimicrobial Agents in 2012 for all medical institutions [15]. Moreover, China’s National Action Plan to Contain Antimicrobial Resistance (2016–2020) was released in 2016 [16]. As the largest producer and consumer of antibiotics in the world [17], China still faces a serious AMR problem [18]. Antibiotic usage in China was almost five-times as high as that in the United States and Europe in terms of daily doses per 1000 inhabitants per day (DID) [19]. Undoubtedly, China must take further steps to curtail overuse of antibiotics and minimize the potential harms, accompanied by periodic antibiotic use population surveys in the community to monitor the effectiveness of these steps. 

Although many measures have focused on antibiotic provision, including clinician training and financial compensation, few patient or resident-oriented interventions have been reported in China [20]. Compared with the convenient location and timely access of the pharmacies, limited availability of physicians coupled with long waiting times in hospitals drives up self-purchasing of antibiotics, particularly in rural areas [21,22]. In this context, there has been easy access to antibiotics without prescription, especially in rural China. Community perceptions of antibiotic use and AMR are very important for their control [23]. Moreover, while COVID-19 has changed our world greatly, including higher rates of antibiotic resistance, as reported in several related studies [24,25], antibiotic use and access patterns of the community population in China during the pandemic are unclear. Therefore, this study aimed to understand antibiotic use and access patterns and their influencing factors among the rural community population in Eastern China between July and August 2020. This was conducted as part of a wider ongoing research program on the burden of antibiotic resistance in China.

## 2. Results

### 2.1. Study Participants

Of the 1379 eligible subjects, including 610 (44.2%) from Village A and 769 (55.8%) from Village B, more than half were female in the age range of 45–64 years. Most of them were married, with under nine years of education, and were blue-collar workers. Nearly half had an average annual household income per capita over 50,000 RMB (US $7470), and over half of them reported one or more chronic diseases. Two-fifths were classed as having a high level of antibiotic knowledge. Compared to Village A, residents in Village B had statistically higher proportions of female, blue-collar and high antibiotic knowledge level, but lower proportions of 45–64 years, under nine years of education and annual income over 50,000 (Table 1).

### 2.2. Distribution and Determinants of Most Recent Antibiotic Use

**Figure 1:**
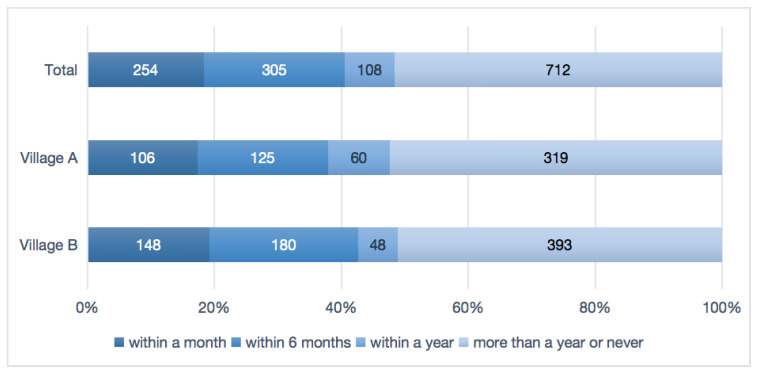
The distribution of last antibiotic use.

Among all the 1379 participants, nearly half (667; 48.4%) reported having taken antibiotics in the last year. The proportions of antibiotic use were 18.4% in the past month and 40.5% within the past six months, respectively. No statistical difference was observed between the two villages, as shown in Figure 1 (*p* > 0.05).

As Table 2 shows, antibiotic use varied over different sociodemographic characteristics, with those in the age range 45–64 years or being married having higher levels of antibiotic use in the past year. No significant differences existed for sex, education, occupation, income, chronic disease history, antibiotic knowledge level and location.

### 2.3. Antibiotic Access and Its Determinants

The majority (59.9%) of participants obtained antibiotics from medical facilities with a prescription. Almost one fifth (17.7%) of respondents obtained antibiotics from pharmacies, and over one fifth (22.4%) reported obtaining antibiotics from other sources outside medical facilities and pharmacies. Among the other sources, the proportion of online purchasing was the highest in this survey (45.0%,139/309). Higher proportions of participants who obtained antibiotics from medical facilities (with prescription) were seen among those who were married, with education levels of under nine years, white-collar workers, with per capita household income over 50,000 and a high level of antibiotic knowledge, without chronic disease history, and lived in Village A (Table 3). 

After adjustment for possible covariates, residents aged 15–44 years, who were blue-collar workers, had an annual household income per capita of less than 10,000 RMB, or a low level of antibiotic knowledge, were significantly more likely to obtain antibiotics from pharmacies, while those with under nine years of education tended to choose medical facility access compared to those with a higher level of education. Those who were unmarried, unemployed, or blue-collar workers, with a low level of antibiotic knowledge or without a history of chronic disease were more likely to obtain antibiotics from other sources rather than medical facilities (Table 4).

## 3. Discussion

Antibiotic resistance is one of the biggest threats worldwide and antibiotic misuse is accelerating the process. Since consumers play an important role in antibiotic use [26], improving public awareness of AMR may help to reduce the indiscriminate use of antibiotics. In this community level study among rural residents in two counties of Eastern China, although they had similar proportions of antibiotic use in the last year, there was a significant difference in where antibiotics were obtained by residents, presumably due to the varied economic and cultural environment between Village A and Village B. Therefore, profiling antibiotic use practices across provinces in China provides valuable information and more attention needs to be paid to context in the development of local measures to improve antibiotic use. To our knowledge, this is the first study to explore antibiotic use behaviors from the perspective of rural Chinese residents during the COVID-19 pandemic. 

In this study, nearly half of the residents reported having used antibiotics within the past 12 months in July 2020, which is in line with results reported by other Chinese studies [27,28]. The proportions of those using antibiotics in the last six and 12 months in this study was substantially lower than that reported by the WHO for China in 2015 (18.4% vs. 28%, 40.5% vs. 57% and 48.4% vs. 71%, respectively) [23]. Even higher rates were observed worldwide, with over three quarters (77%) reporting antibiotic use within a year [23]. Those findings imply that China has made some achievements in rationalizing antibiotic use. However, the rate of antibiotic use in this study was still higher than the WHO’s recommendation of 30% [29]. Marriage and middle age were found to be potential factors related to higher levels of antibiotic utilization, partly because interactions between relatives and friends may foster a culture of antibiotic use and provide more chances of antibiotic exposure within this population. This suggests a need for specific community-level interventions on antibiotic use for certain sociodemographic groups in rural China. Notably, socioeconomic status and antibiotic knowledge level do not have a significant influence on antibiotic use; this may be explained by the fact that symptoms are strong determinants of antibiotic use, with the same incidence of viral diseases such as influenza contributing to similar usage rates [23,28]. 

Since 2011, accompanied by new healthcare reforms in China, many measures have been effectively implemented to ensure the appropriate use of antibiotics in public hospitals under the surveillance system [20]. However, although the existing guidance [16] proposes that prescription-only selling in retail pharmacies would achieve full coverage by 2020, easy access to non-prescription antibiotics from pharmacies has been commonly observed in most areas of China [26]. In the cultural context of antibiotic over-prescribing, those with health insurance that requires co-payment and those with limited AMR knowledge or low health literacy have tended to self-medicate and may exert invisible pressure on the staff of medical facilities and drugstores to obtain antibiotics for themselves or their families [16,30,31,32]. High levels of over-the-counter antibiotic dispensing and self-medication need to be addressed. 

Our study findings on the preferred sources for obtaining antibiotics generally reflects wider buying habits for antibiotics in the population. A multi-country survey in 2015 revealed that most respondents (81%) obtained antibiotics from a doctor or nurse, and the proportion was relatively consistent across the countries surveyed, which was 74% in China [23]. In the current study, the proportion of antibiotics obtained from medical facilities was 59.9%, and over one fifth were from other sources when they were last obtained, corroborating the findings of previous studies that China had the highest proportion of sources outside medical facilities and pharmacies [6]. The multi-country study stated that China was the only country apart from India in which respondents (5%) reported accessing antibiotics online, and China also has the highest proportion of respondents (4%) who reported obtaining antibiotics from friends or family members [23]. Well-enforced regulations are clearly important to restrict outpatient antibiotic availability, including electronic prescriptions issued by online “doctors” who may lack medical qualifications [33]. The higher proportion of antibiotics being accessed via medical facilities in Village A may partly relate to stricter enforcement and supervision of prescription-only policies at pharmacies in Village A than Village B, indicating that modifying access to antibiotics should not only be conducted at the individual level of residents’ behavior, but also through strengthening policy implementation and supervision at the local government level. 

The distribution of antibiotic acquisition also varied greatly across sociodemographic groups in this study. Compared with older people, young and middle-aged groups preferred to obtain antibiotics over the counter from pharmacies rather than via clinical consultations at medical facilities, which is contrary to findings from Central China [26]. This discrepancy may be attributed to variations in local culture, impact of the pandemic and sampling frames and sizes in different studies. Further investigations are warranted to elucidate these differences. In our study, those who were not married tended to choose other sources to obtain antibiotics, perhaps because unmarried people are more likely to be younger adults living with relatives or friends who provide them with antibiotics as an aspect of care. This finding implies that more attention should be paid to this group in community settings. Moreover, consistent with findings from European countries and Southeast Asia [34,35], those in blue collar occupations and with low incomes are the least likely to have the spare time to spend queuing in medical facilities and most cannot afford to take time off, so they obtain antibiotics from other sources as a “quick fix” to enable them to continue working.

In China, antibiotics are believed to be a panacea and are mistakenly taken for prophylaxis by the general public with limited medical knowledge [15]. Supported by several studies [28,36,37], our study also found that those with a low level of antibiotic knowledge had an increased tendency to obtain antibiotics outside medical facilities. On the other hand, it must be mentioned that well-educated residents were more prone to choose retail pharmacies to access antibiotics rather than medical facility attendance. One possible reason is that better educated people tended to seek outside care and self-medicate with antibiotics, aligning with the multi-country survey of the WHO [23]. This interesting paradox suggests that simple knowledge improvement may not be enough to improve their behaviors in antibiotic use and access. In addition, subjects with some chronic diseases tended to obtain antibiotics from other sources in this study. A possible explanation is that these people, being less healthy overall, are more likely to have existing supplies of drugs, including antibiotics, at home, which they can reuse. Therefore, developing interventions targeted to and tailored for specific residents is critical.

Antibiotic over-prescription was previously thought to be more prevalent in the less developed areas of China [38], but this study indicates that there were high proportions of antibiotic access without prescription among rural community residents even in two Eastern provinces that are among China’s most developed areas, at least during the pandemic. This study highlights the urgent need for multifaceted antibiotic stewardship programs in rural Chinese community healthcare settings and pharmacies. The public should be provided with clear information on antibiotic use and AMR control, including advice such as only using antibiotics with a prescription by certified health professionals and never to share or use left-over antibiotics [23]. Additionally, professional staff training is essential to eliminating non-prescription antibiotic dispensing at community pharmacies. In addition, national and local health departments in China should further strengthen the supervision of health care providers, especially from pharmacies and internet channels [32,39], where one of the effective surveillance measures may be the potential use of mobile technology to monitor dispensing. 

Of course, there were some inevitable limitations in this study. One was that the cross-sectional study design has very limited ability in causal inference. Another was that the self-reported survey may lead to a degree of bias, with respondents providing the answer they believe is “expected”. Additionally, a notable fact is that seasonal fluctuations in morbidity affect the need for medicines, which may underestimate or overestimate the proportions of antibiotic use over one month or over the course of six months during the pandemic [40]. 

## 4. Materials and Methods

### 4.1. Study Design and Population

A cross-sectional survey was conducted with a sample of the rural Chinese population in Eastern China between July and August 2020. Based on the per capita gross domestic product and antibiotic application in industry and agriculture, the research was carried out in Zhejiang and Jiangsu provinces. Given the antibiotic use rate of 50% in China over one year, the minimum sample size was estimated to be 400 with a 5% margin of error. Using the rapid cluster sample survey methodology recommended by the WHO [41], one village was purposively selected as the study site in each province (Village A of Zhejiang Province and Village B of Jiangsu Province), and a total of 1494 residents were recruited from both villages into the study. The inclusion criteria were as follows: (1) aged 15 years old and over; (2) who had lived in the study sites for no less than six months; and (3) consenting to inclusion in the study. 

This study received ethical approval from the Institutional Review Board of School of Public Health, Fudan University, China (number IRB#2019-03-0733). Written informed consent was obtained from all participants prior to any data collection. Questionnaires which had more than three of the knowledge items unfinished were excluded from further analysis (*n* = 20). Five individuals with missing sex variables, and 90 without socioeconomic answers were also excluded. A final total of 1379 participants were included in the analysis. 

### 4.2. The Questionnaire and Data Collection

A structured questionnaire was used by trained investigators (based on the WHO “Antibiotic Resistance: Multi-Country Public Awareness Survey” [23] and adapted after a pilot study with 523 adults in 2019). Detailed information from respondents including demographic information, chronic disease history, antibiotic use behaviors, and antibiotic knowledge was collected face-to-face using a structured electronic questionnaire via the “wenjuanxing” online procedure on electric tablets. Demographic information included sex, age, ethnicity, marriage status, education, occupation, and annual household income per capita. Chronic disease history covered nine common chronic diseases. Antibiotic use included the use of antibiotics, whether obtained with a prescription, and most recent use; respondents were asked if they had last taken antibiotics in the past month, 2–6 months, 7–12 months, over one year ago or never, and antibiotic use was defined as yes if last reported antibiotic use was within the past year. Options for antibiotic access included: inpatient/outpatient service in medical facilities (including village clinics, township healthcare centers, and hospitals) with prescription; medical stores or pharmacies; and others (including friends or family members, online, stalls or hawkers, saved up from a previous time, and somewhere or someone else). 

Antibiotic knowledge consists of attitude to and knowledge of antibiotics and antibiotic resistance and was assessed based on a three-point scale (yes, no, uncertain) as well as a five-point Likert scale (strongly disagree, disagree, uncertain, agree, strongly agree). 

### 4.3. Analysis 

For the antibiotic knowledge items, answers were coded as correct based on WHO recommendations, with each correct answer assigned a score of “1” and other responses (i.e., incorrect or unsure response) a score of “0”. In the five-point section, responses were given a corresponding score of 5, 4, 3, 2 and 1 for positive response to negative response [42]. Then, the comprehensive score (with the full score of 122) of antibiotic knowledge level was calculated and categorized into low, medium, and high levels by the quantile method for further analysis.

Descriptive statistics were performed on the whole dataset and reported as frequency and percentages for qualitative variables. The Chi square test was performed to primarily explore the possible determinants of antibiotic use and its access. The multinominal logistic regression analysis was further performed to identify factors significantly associated with antibiotic access. In this multinomial model, access to antibiotics via medical facilities was used as the reference level. Adjusted odds ratios (adjusted ORs) and 95% confidence intervals (95% CIs) were estimated. The level of statistical significance was set at *p* < 0.05 and all analyses were performed in R software (Version 4.0.3; R Foundation for Statistical Computing, Vienna, Austria).

## 5. Conclusions

In summary, the antibiotic use behavior of rural community residents in Eastern China is suboptimal. Our findings help to address the scarcity of empirical evidence of antibiotic use behaviors. China still needs to make considerable efforts to effectively contain AMR, such as by constructing a supportive and supervised environment for rational antibiotic use and strengthening antibiotic information dissemination for the rural population to limit over-the-counter antibiotics. Given the complex relationship observed in this and other studies between education, economic status and antibiotic self-medication, further research into the reasons for non-prescribed antibiotic use in specific population groups would help to inform the development of appropriate and effective targeted interventions.

## Figures and Tables

**Table 1:** General characteristics of rural residents in Eastern China by area.

Variables	n (%)			χ2	*p*-Value
	Total	Village A	Village B		
Total	1379	610 (44.2)	769 (55.8)		
Sex				5.456	0.023
Female	774 (56.1)	321 (52.6)	453 (58.9)		
Male	605 (43.9)	289 (47.4)	316 (41.1)		
Age (years)				14.679	0.001
15–44	97 (7.0)	49 (8.0)	48 (6.2)		
45–64	658 (47.7)	320 (52.5)	338 (44.0)		
>65	624 (45.3)	241 (39.5)	383 (49.8)		
Married				1.111	0.292
Yes	1198 (86.9)	537 (88.0)	661 (86.0)		
No	181 (13.1)	73 (12.0)	108 (14.0)		
Education years				31.631	<0.001
0–9	829 (60.1)	418 (68.5)	411 (53.4)		
>9	550 (39.9)	192 (31.5)	358 (46.6)		
Occupation				143.158	<0.001
Unemployed	279 (20.2)	195 (32.0)	84 (10.9)		
Blue-collar	951 (69.0)	319 (52.3)	632 (82.2)		
White-collar	149 (10.8)	96 (15.7)	53 (6.9)		
Annual household income per capita, RMB				179.390	<0.001
≤10,000	312 (22.6)	67 (11.0)	245 (31.9)		
10,001–30,000	210 (15.2)	56 (9.2)	154 (20.0)		
30,001–50,000	232 (16.8)	95 (15.6)	137 (17.8)		
>50,000	625 (45.3)	392 (64.3)	233 (30.3)		
Chronic disease history				3.007	0.074
Yes	756 (54.8)	318 (52.1)	438 (57.0)		
No	623 (45.2)	292 (47.9)	331 (43.0)		
Antibiotic knowledge level				16.547	<0.001
Low	263 (28.6)	197 (32.3)	66 (21.2)		
Medium	299 (32.5)	200 (32.8)	99 (31.8)		
High	359 (39.0)	213 (34.9)	146 (46.9)		

**Table 2:** Determinants of antibiotic use in past one year among rural residents in Eastern China.

Variables	Antibiotic Use [n (%)]		χ^2^	*p*-Value
	Yes	No		
Total	667 (48.4)	712 (51.6)		
Sex			0.443	0.506
Female	381 (49.2)	393 (50.8)		
Male	286 (47.3)	319 (52.7)		
Age (years)			8.612	0.013
15–44	38 (39.2)	59 (60.8)		
45–64	343 (52.1)	315 (47.9)		
≧65	286 (45.8)	338 (54.2)		
Married			5.766	0.016
Yes	595 (49.7)	603 (50.3)		
No	72 (39.8)	109 (60.2)		
Education years			0.146	0.702
0–9	397 (47.9)	432 (52.1)		
>9	270 (49.1)	280 (50.9)		
Occupation			0.155	0.926
Unemployed	133 (47.7)	146 (52.3)		
Blue-collar	460 (48.4)	491 (51.6)		
White-collar	74 (49.7)	75 (50.3)		
Annual household income per capita, RMB			3.377	0.337
≤10,000	139 (44.6)	173 (55.4)		
10,001–30,000	105 (50.0)	105 (50.0)		
30,001–50,000	121 (52.2)	111 (47.8)		
>50,000	302 (48.3)	323 (51.7)		
Chronic disease history			2.244	0.134
Yes	380 (50.3)	376 (49.7)		
No	287 (46.1)	336 (53.9)		
Antibiotic knowledge level			1.913	0.384
Low	122 (46.4)	141 (53.6)		
Medium	155 (51.8)	144 (48.2)		
High	183 (51.0)	176 (49.0)		
Area			0.148	0.700
Village A	291 (47.7)	319 (52.3)		
Village B	376 (48.9)	393 (51.1)		

**Table 3:** Determinants of antibiotic access last time among rural residents in Eastern China.

Variables	Antibiotic Access [n (%)]			χ^2^	*p*-Value
	Medical Facilities (with Prescription)	Medical Stores or Pharmacies	Others		
Total	826 (59.9)	244 (17.7)	309 (22.4)		
Sex				2.021	0.364
Female	453 (58.5)	137 (17.7)	184 (23.8)		
Male	373 (61.7)	107 (17.7)	125 (20.7)		
Age (years)				4.493	0.343
15–44	57 (58.8)	23 (23.7)	17 (17.5)		
45–64	403 (61.2)	113 (17.2)	142 (21.6)		
≧65	366 (58.7)	108 (17.3)	150 (24.0)		
Married				6.788	0.034
Yes	726 (60.6)	217 (18.1)	255 (21.3)		
No	100 (55.2)	27 (14.9)	54 (29.8)		
Education years				6.383	0.041
0–9	503 (60.7)	130 (15.7)	196 (23.6)		
>9	323 (58.7)	114 (20.7)	113 (20.6)		
Occupation				18.953	0.001
Unemployed	181 (64.9)	34 (12.2)	64 (22.9)		
Blue-collar	539 (56.7)	188 (19.8)	224 (23.5)		
White-collar	106 (71.1)	22 (14.8)	21 (14.1)		
Annual household income per capita, RMB				12.966	0.044
≤10,000	164 (52.6)	72 (23.1)	76 (24.3)		
10,001–30,000	129 (61.4)	35 (16.7)	46 (21.9)		
30,001–50,000	136 (58.6)	43 (18.5)	53 (22.9)		
>50,000	397 (63.5)	94 (15.1)	134 (21.4)		
Chronic disease history				9.574	0.008
Yes	483 (56.7)	162 (19.0)	207 (24.3)		
No	343 (65.1)	82 (15.6)	102 (19.3)		
Antibiotic knowledge level				13.699	0.008
Low	146 (55.5)	43 (16.3)	74 (28.2)		
Medium	198 (66.2)	33 (11.0)	68 (22.8)		
High	248 (69.1)	43 (12.0)	68 (18.9)		
Area				115.560	<0.001
Village A	431 (70.7)	33 (5.4)	146 (23.9)		
Village B	395 (51.4)	211 (27.4)	163 (21.2)		

**Table 4:** Multinomial logistic regression on last antibiotic access among rural residents in Eastern China.

Variables	Pharmacies		Others	
	OR (95% CI)	*p*-Value	OR (95% CI)	*p*-Value
Sex (Ref: Female)				
Male	0.95 (0.75~1.21)	0.688	0.85 (0.70~1.03)	0.100
Age (Ref: ≧65 years)				
15–44	2.21 (1.28~3.81)	0.004	0.70 (0.43~1.14)	0.151
45–64	1.17 (0.87~1.59)	0.298	0.91 (0.72~1.17)	0.472
Married (Ref: Yes)				
Other	0.70 (0.48~1.02)	0.065	1.42 (1.06~1.89)	0.018
Occupation (Ref: White-collar)				
Unemployed	0.94 (0.59~1.49)	0.776	1.74 (1.19~2.54)	0.005
Blue-collar	1.51 (1.01~2.26)	0.044	1.96 (1.38~2.77)	<0.001
Education (Ref: >9 years)				
0–9	0.64 (0.49~0.83)	0.001	0.96 (0.77~1.20)	0.725
Annual household income per capita (Ref: >50,000 RMB)				
≤10,000	1.91 (1.34~2.73)	0.001	1.10 (0.82~1.48)	0.533
10,001–30,000	1.10 (0.76~1.61)	0.615	0.92 (0.68~1.25)	0.587
30,001–50,000	1.25 (0.89~1.76)	0.201	1.05 (0.79~1.38)	0.753
Antibiotic knowledge level (Ref: High)				
Low	2.74 (2.01~3.72)	<0.001	1.50 (1.18~1.92)	0.001
Medium	1.07 (0.74~1.57)	0.713	1.23 (0.93~1.62)	0.143
Chronic disease history (Ref: Yes)				
No	1.23 (0.97~1.58)	0.093	1.76 (1.44~2.16)	<0.001

Note: The medical facility access was used as the reference level.

## Data Availability

The data presented in this study can be made available upon request from the corresponding author. The dataset is not publicly available as it forms part of ongoing research and analysis. It will be made publicly available once the major study publications are completed.
